# Massive acetabular bone loss: Limits of trabecular metal cages

**DOI:** 10.4103/0019-5413.73664

**Published:** 2011

**Authors:** Manuel Villanueva-Martínez, Antonio Ríos-Luna, Juán Diaz-Mauriño

**Affiliations:** Hospital General Universitary Gregorio Marañón, Madrid; 1 Hospital Virgen del Mar, University of Almeria, Almería

**Keywords:** Bone ingrowth, massive acetabular bone loss, trabecular metal cups

## Abstract

Massive acetabular bone loss (more than 50% of the acetabular area) can result in insufficient native bone for stable fixation and long-term bone ingrowth of conventional porous cups. The development of trabecular metal cages with osteoconductive properties may allow a more biological and versatile approach that will help restore bone loss, thus reducing the frequency of implant failure in the short-to-medium term. We report a case of massive bone loss affecting the dome of the acetabulum and the ilium, which was treated with a trabecular metal cage and particulate allograft. Although the trabecular metal components had no intrinsic stability, they did enhance osseointegration and incorporation of a non-impacted particulate graft, thus preventing failure of the reconstruction. The minimum 50% contact area between the native bone and the cup required for osseointegration with the use of porous cups may not hold for new trabecular metal cups, thus reducing the need for antiprotrusio cages. The osteoconductive properties of trabecular metal enhanced allograft incorportation and iliac bone rebuilding without the need to fill the defect with multiple wedges nor protect the reconstruction with an antiprotrusio cage.

## INTRODUCTION

Massive acetabular bone loss is defined as bone loss affecting more than 50% of the acetabular cup, with distortion of acetabular geometry, column damage, and pelvic discontinuity. Surgical options include massive structural grafts, oblong revision cups, custom triflanged cages, noncustom reconstruction rings, cages with modular porous metal augments, and impaction grafting techniques.^1^,^2^

Reconstructive cages are the most commonly used devices, but they do not allow for biological fixation, thus leading to failure of the construct in the medium-to-long term.

Modular trabecular metal cages could be a better option for biological fixation than the conventional systems. Trabecular metal has high volumetric porosity (70 – 80%), a high friction coefficient (40 – 75% greater than the standard porous coatings), and a low modulus of elasticity, which allows for more physiological load transfer. Therefore, less initial contact with the native bone is required to ensure stability, and the high osteoconductive capacity of the trabecular metal facilitates biological fixation^3^–^6^ and restoration of the bone mass in cases of massive acetabular bone loss, which is related to implant survival in the medium term. Our case illustrates both the limits of bone-implant contact to get osseointegration and the limits of osteconductive capacity with trabecular metal cages.

## CASE REPORT

A 64-year-old woman with developmental dysplasia of the hip had undergone surgery on both hips. Fifteen years ago she underwent varus osteotomy of the right hip and 11 years ago she underwent total hip arthroplasty of the left hip, in which a 62-mm cup (Multilock, Zimmer^®^) with a high rotation center was fixed with screws.

**Figure 1:**
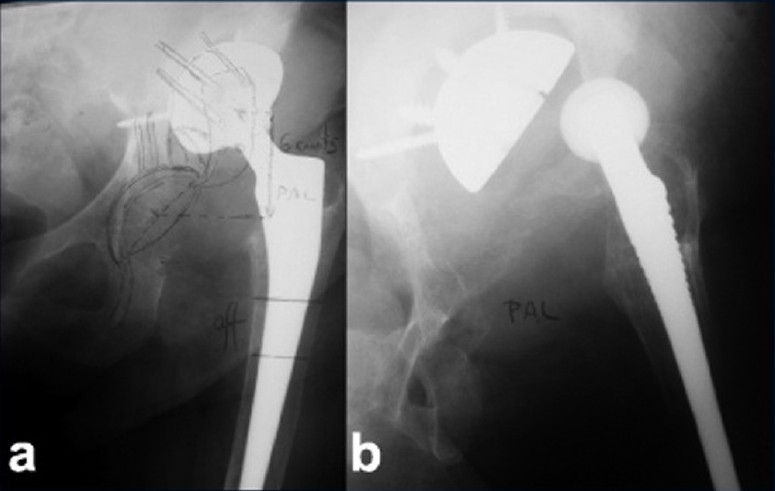
Anteroposterior radiograph of the left hip showing (a) elevation of the center of rotation by 6 cm (preoperative planning). (b) X-ray (anteroposterior view) showing dislocated hip, massive proximal and acetabular bone loss

She was walking pain free until three years ago. Then gradually pain around hip started. The cup was found to have loosened, migrating proximally with massive osteolysis of the ilium. The patient also had an unstable and painful right knee arthroplasty (Insall-Burstein II, Zimmer^®^), probably due to malpositioning, overstress, and limb length discrepancy. For the last 18 months of this period she was only able to stand up and take a few assisted steps. Surgery was scheduled, but a fall at home led to a periprosthetic fracture of the right knee that was treated with a Liss plate (Synthes^®^). Six months later, when the periprosthetic fracture had consolidated, hip surgery was again scheduled. Prior to surgery, the patient was admitted urgently due to dislocation of the hip prosthesis, with a shortening of 6 cm and deformity of the limb [Figure 1].

We considered the following options. Massive structural allograft protected by an antiprotrusio cage. Large trabecular metal cup plus allograft protected by an antiprotrusio cage (‘cup-cage’ construct). A small cup, housed in the remaining native acetabulum, with acetabular protrusio or cotyloplasty plus allograft. We also considered the possibility of stem removal and femoral shortening osteotomy if the sciatic nerve function was affected upon restoration of the center of rotation. During surgery, the bone loss was seen to affect the entire superior and anterior iliac area, leaving a thin posterior wall that was proximal to the native acetabulum. It had bulged, and was sclerotic and distorted as a result of osteolysis and the presence of the previous cup. The available femoral allograft was insufficient to fill the bone loss and there was no superior or medial bone stock to which it could be fixed. The Burch-Schneider cage was too short to reach any healthy proximal bone where it could be fixed to bridge the affected area. The second option, the ‘cup-cage’ construct, would also have implied drilling more bone only to achieve poor lower contact and no upper or anteroposterior support.

**Figure 2:**
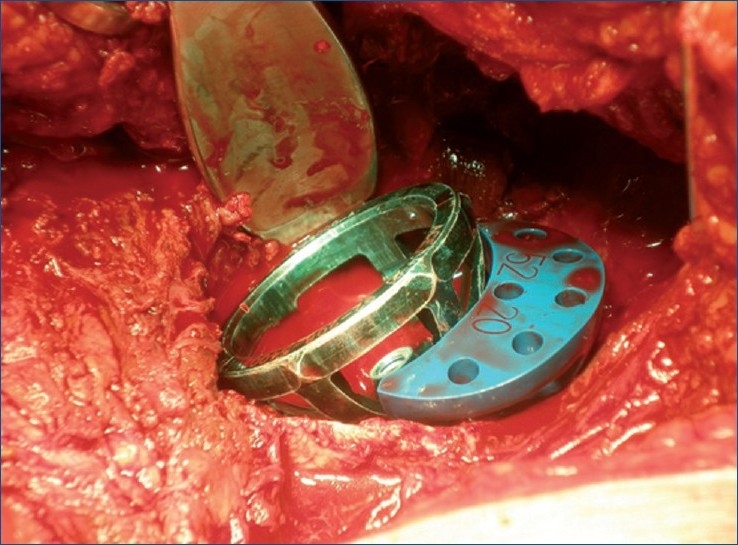
Peroperative photograph showing massive iliac bone loss. Trial components on the posterior iliac rim. No intrinsic stability

**Figure 3:**
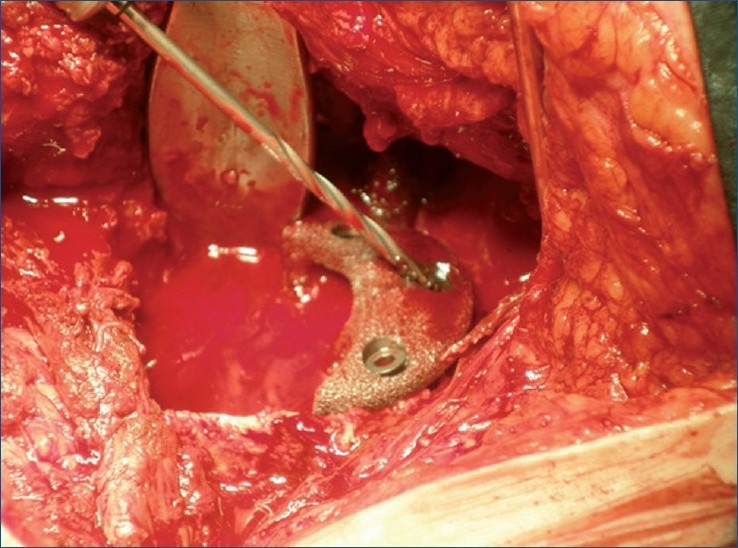
Peroperative photograph showing tantalum wedge screwing. The augment is drilled to redirect the screws to an area with remnant bone

**Figure 4:**
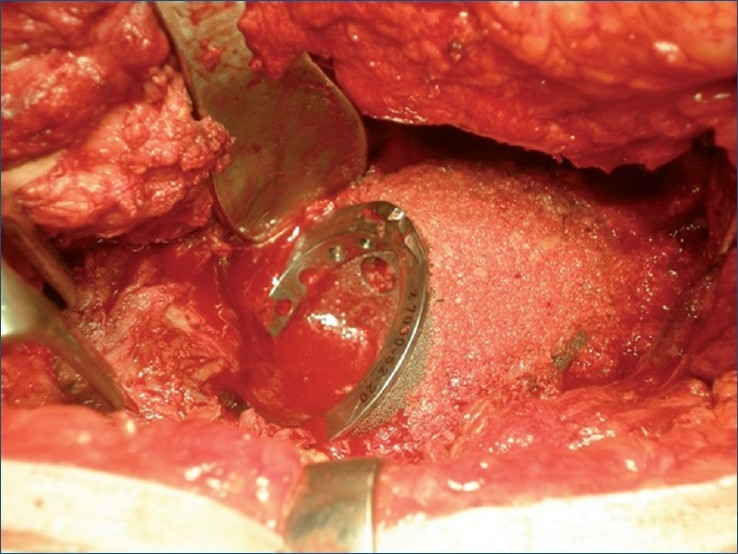
Peroperative photograph showing particulate graft filling the upper bone loss and covering the wedge

We used a high-speed drill to refresh the posterior and upper sclerotic rim, and we fixed the augment to the adapted area by drilling the trabecular metal, to redirect the screws in search of remnant bone [Figures 2 and 3]. A small trabecular metal cup was positioned (Trilogy, Zimmer^®^) in the native cup — the only area with remnant bone — resulting in minimum contact with the lower section. Neither the wedge nor the cup presented any intrinsic stability. Both components had to be held in place with Kocher clamps, while they were being drilled. The proximal wedge was screwed against the thin medial wall of the defect at the posterior superior ilium, with no intrinsic stability, little purchase for the screws (two for the cup and two for the wedge), and a slight residual motion of the wedge and cup after insertion of the screws. The massive bone loss was filled with particulate allograft material, and the proximal wedge was covered [Figure 4] and protected with a layer of Surgicel (Johnson and Johnson^®^). The liner was placed into the trabecular metal cup and a trial reduction to check for stability was performed. At this time, we carried out a ‘wake-up test’ to check the sciatic nerve function after lowering the center of rotation to more than 5 cm.

**Figure 5 (a and b):**
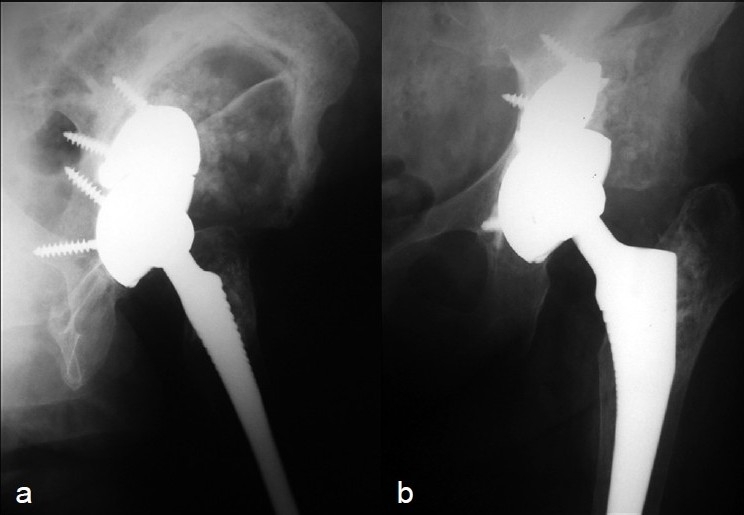
X-ray (anteroposterior view) showing progression of allograft incorporation

Weight bearing was avoided for three months. At the end of this period, before ambulation was allowed, the Liss plate was removed, and the unstable contralateral total knee arthroplasty was repaired with a condylar constrained prosthesis (Nex-Gen LCCK, Zimmer^®^). The patient started rehabilitation after more than two years without independent walking. and over nine months without standing-up. She started by maintaining a standing position, followed by gradual ambulation. Four years later, the patient can walk without crutches or canes, her knee is stable, her hip painless, and the massive upper bone loss has filled [Figures 5a and 5b]. She uses a 1-cm right heel lift, and her Harris hip score is 85 points (0 – 100).

## DISCUSSION

The main disadvantage of the conventional antiprotrusio cages is the absence of a porous coating for biological fixation. Custom reconstruction cages offer the possibility of mechanical and biological fixation (they have a porous coating). Cost considerations, lack of availability, intraoperative versatility, and the fact that most of the bone loss is filled with metal, have limited their use.^2^

In cases where more than 50% of the cup has to be supported by the allograft, structural allografts must be protected with a reconstruction cage in order to avoid high late failure rates,^7^,^8^ as a result of slow and incomplete incorporation. In our case, the defect, which was aggravated by osteolysis and previous high cup placement, involved two-thirds of the height of the iliac wing; therefore, neither the cage nor the allograft would have reached the upper part of the ilium and could not have been fixed to the very thin remaining iliac bone.

The second option, the ‘cup-cage’ construct, required more bone to be reamed to insert a larger cup, thus increasing bone loss and leaving the cup even more uncovered with native bone. In addition, the cage failed to reach a healthy upper area for the screws to be anchored. We therefore ruled out this option.^9^

The third option was to insert a small tantalum cup in the native dysplastic acetabulum. The contact area between the native bone and the cup was much less than 50% and the construct had no intrinsic stability as the screws had little purchase.

Despite the lack of initial stability, the properties of the trabecular metal favored graft incorporation and osseointegration, thus avoiding failure of the reconstruction in the short term, when most mechanical failures are observed with other systems.

In the setting of massive acetabular bone loss the surgeon has the option of filling the defect with multiple trabecular metal wedges rather than filling it with a particulate graft, as we finally did. Although the trabecular metal augments may act as structural grafts, bone stock restoration must be compromised by a large amount of metal. We achieved our goal of restoring bone mass, which was related to implant survival in the short-to-medium term, and the combination of the trabecular metal cup and particulate graft proved to be reliable. Impacted particulate cancellous bone allows rapid vascular invasion and complete and uniform incorporation.^10^ In this case the massive iliac bone loss was regenerated despite not performing a standard ‘impaction grafting technique’.

Trabecular metal cages may enable better mechanical fixation and subsequent biological fixation, with the purpose of improving long-term implant survival.^3^,^11^–^13^ The low elastic modulus allows more physiological load transmission, with no stress shielding effect and a more normal remodelling pattern. The need for structural grafts or custom implants could be reduced, as the modular wedges in turn acted as structural allografts, without the reabsorption risk usually associated with the latter.^14^–^17^ In addition, the highly conductive properties of tantalum facilitate bone ingrowth within the trabeculae.^18^

The minimum 50% contact between the native bone and the cup are considered necessary to achieve biological fixation, and to protect the cup with an antiprotrusio cage may not hold for new trabecular metal cups, and this could reduce the need for antiprotrusio cages,^5^,^16^ as illustrated by this case.
